# Supplementary material for: Rapid identification of microbial pathogens and antimicrobial resistance from bloodstream infections using long-read sequencing
Source: Microb Genom. 2026 Jun 11;12(6):001699. doi: 10.1099/mgen.0.001699 (PMC13256323; doi:10.1099/mgen.0.001699)
Supplement: Supplementary Material 1. [file mgen-12-01699-s001.pdf]

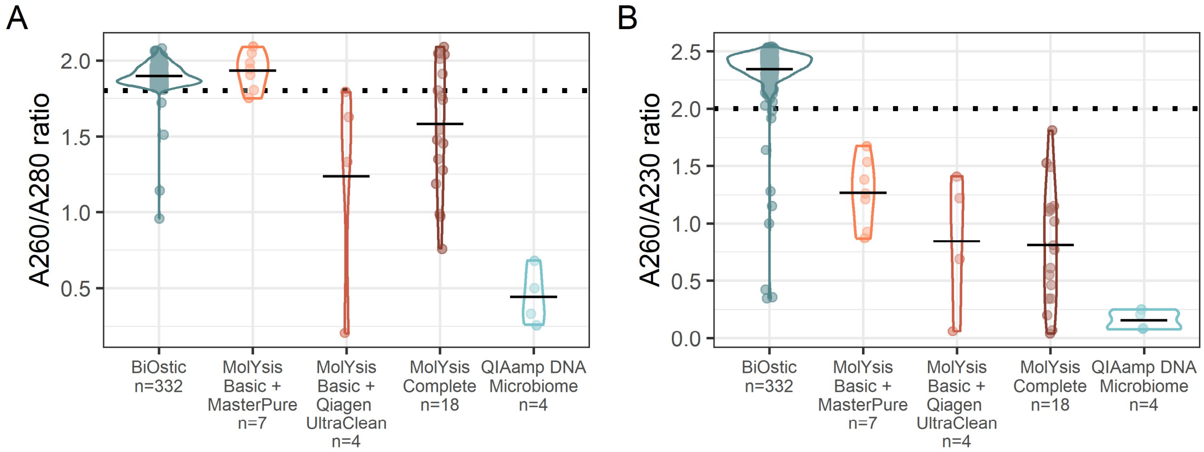

Supplementary Figure 1. DNA purity across different DNA extraction kits for (**A**) A_{260}/A_{230} and (**B**) A_{260}/A_{280} ratios. Dotted lines indicate recommended values for pure DNA. Solid lines indicate means for each kit. No sequencing data was obtained with MolYsis extracts nor with QIAamp extracts due to immediate pore death upon library addition to flow cell.

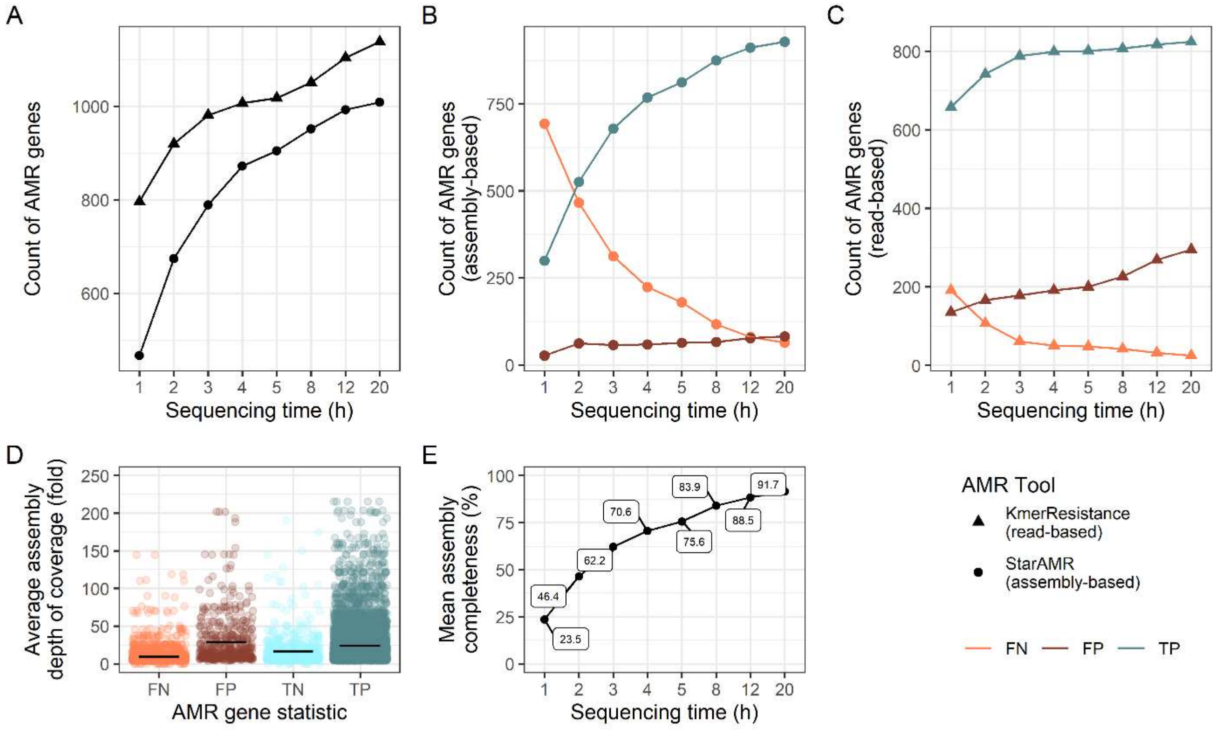

Supplementary Figure 2. Count of correctly-identified AMR genes in 281 positive BCs using the ONT method. (**A**) Count of total AMR genes detected by both read-based and assembly-based tools. (**B**) Count of AMR genes detected by assembly-based tool (StarAMR) and (**C**) read-based tool (KmerResistance). The reference dataset was matching pure isolates sequenced by Illumina and assemblies analyzed for AMR genes with StarAMR and KmerResistance. Note y-axis scales are different for panel B and C due to differences in the number of genes in each database. The total number of AMR genes detected is 992 in assemblies and 849 in reads, and correctly-identified genes include the correct allelic variant. FosB and pbp5 are not included in read-based KmerResistance database and were excluded, and *bla*$_{SHV}$ alleles were also excluded as many do not have described phenotypes in the ResFinder database but depending on the variant can encode resistance to multiple antimicrobials. (**D**) Average assembly depth of coverage for positive or missed detections of AMR genes. Lines represent the mean. (**E**) Mean assembly completeness over sequencing time, which mirrors assembly-based AMR gene detection. FN = false negative (gene missed in ONT method), TP = true positive (gene correctly detected), FP = false positive (gene detected in ONT method that was not detected in Illumina).

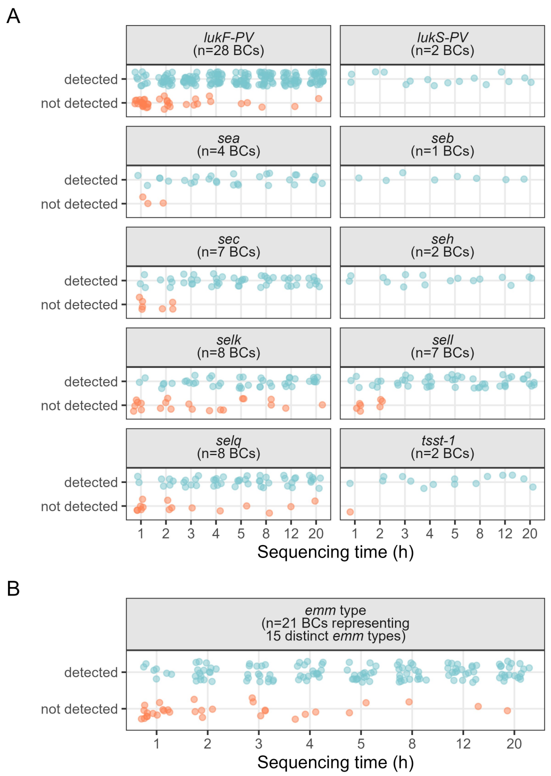

Supplementary Figure 3. Time to detection for **(A)** *S. aureus* toxin genes across 30 positive blood cultures (BCs) and (**B**) *S. pyogenes emm* type across 21 BCs. Each point represents an individual BC.

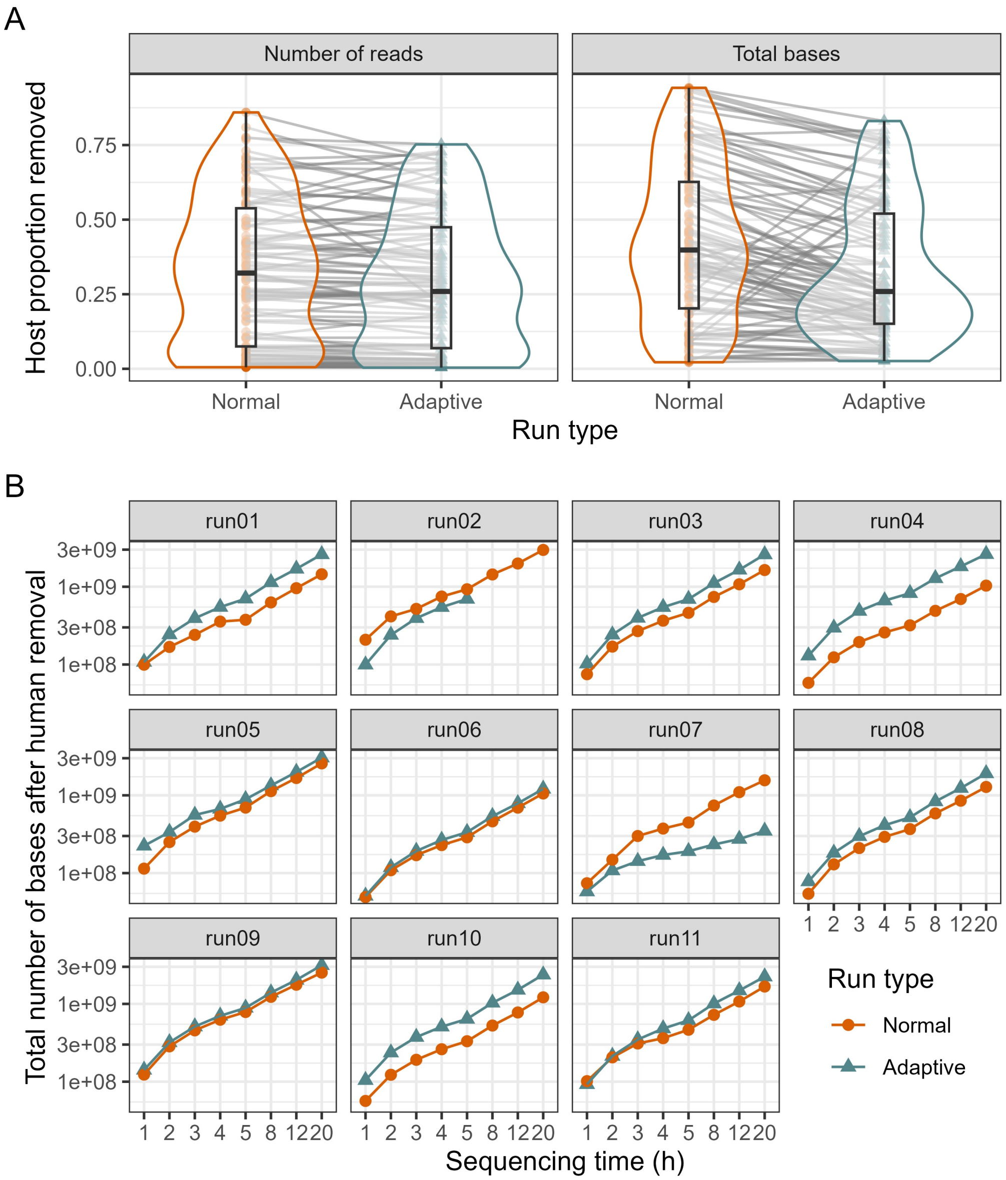

Supplementary Figure 4. (**A**) Number of host reads (left panel) and bases (right panel) removed during analysis in adaptive vs normal (non-adaptive) runs. Lines represent 25th, median, and 75th percentiles. (**B**) Average number of microbial bases sequenced over time (post host depletion) faceted by adaptive run. Each point represents the mean of 4 – 12 BCs.

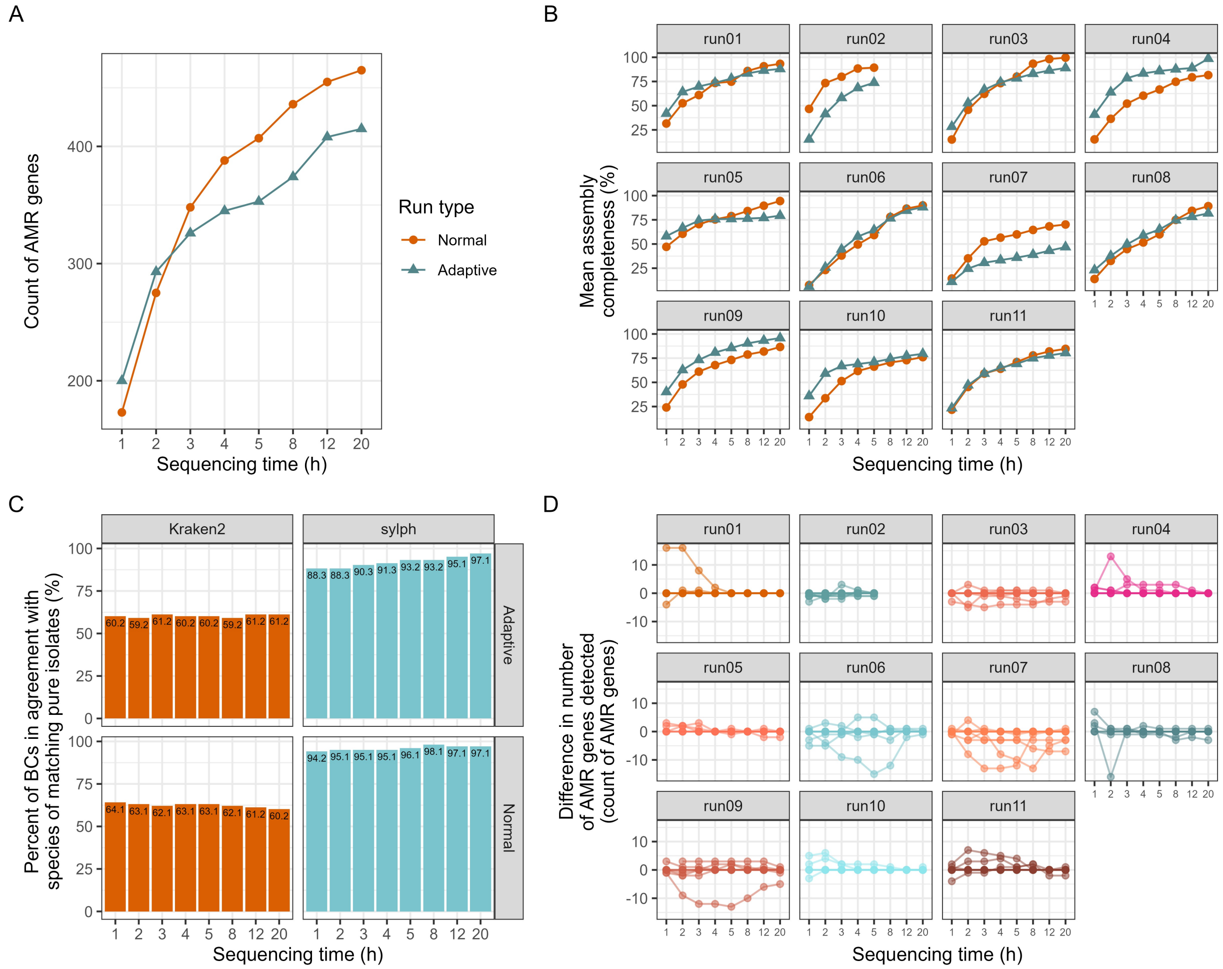

Supplementary Figure 5. Data output by adaptive sampling runs compared to non-adaptive runs. “Normal” indicates non-adaptive sampling whereas “Adaptive” indicates the libraries were sequenced with host depletion. (**A**) The total number of AMR genes detected over time by assembly-based tool (StarAMR) across all runs for normal and adaptive runs. (**B**) Difference in the number of AMR genes detected across all samples in each run, faceted by each run (n=11). Values above 0 indicate more AMR genes in adaptive data and values below 0 indicate more AMR genes in non-adaptive/normal data. (**C**) Proportion of BCs in agreement with genus (top panel) and species (bottom panel) identity of matching pure isolates over time using Kraken2 (left panel) and sylph (right panel). (**D**) Mean assembly completeness over sequencing time faceted by run. Each run contained between 4 – 12 BCs.
